# The immediate effects of a brief gratitude intervention on positive affect, negative affect, and heart rate: a randomized controlled study

**DOI:** 10.3389/fpsyg.2026.1955454

**Published:** 2026-09-23

**Authors:** Dilara Suru

**Affiliations:** Department of Psychology, Halic Üniversitesi, Beyoğlu, Türkiye

**Keywords:** gratitude intervention, heart rate, negative affect, positive affect, positive psychology, randomized controlled trial

## Abstract

**Background:**

Gratitude interventions have consistently been associated with improvements in psychological well-being; however, their immediate physiological effects remain less well understood. Although previous studies have demonstrated beneficial affective outcomes, evidence regarding cardiovascular responses, particularly heart rate (HR), has been inconsistent because of methodological heterogeneity and the use of different physiological indicators. It was hypothesized that a brief gratitude intervention would increase positive affect and decrease negative affect relative to an active control condition, and would produce a different pattern of HR responding than the active control condition, without a directional prediction for HR.

**Objective:**

The present study examined the immediate effects of a brief gratitude intervention on positive affect, negative affect, and HR among university students.

**Methods:**

A randomized controlled pretest–posttest experimental design was employed with 41 undergraduate students recruited from a university in Türkiye. Participants were randomly assigned to either a gratitude-writing intervention (*n* = 21) or an active neutral-writing control condition (*n* = 20). Positive and negative affect were assessed with the Positive and Negative Affect Schedule (PANAS; Watson et al., 1988) before and after the intervention, while HR was measured repeatedly across five occasions of the experimental session using a fingertip pulse oximeter. Data were analyzed using mixed-design analyses of variance (ANOVA), baseline-adjusted analyses of covariance (ANCOVA), and complementary nonparametric sensitivity analyses.

**Results:**

Participants who completed the gratitude intervention demonstrated significantly greater increases in positive affect and greater reductions in negative affect than the control group; these findings were supported by mixed ANOVA and baseline-adjusted ANCOVA models. No significant differences were observed between groups in HR across the experimental session. The hypotheses concerning affective outcomes were therefore supported, whereas the hypothesis regarding HR was not.

**Conclusion:**

A brief gratitude intervention produced immediate improvements in self-reported affect but did not produce a detectable change in HR specifically. Because HR is only one narrow index of cardiovascular activity, this null result should not be generalized to “physiological effects” as a whole; more sensitive or theoretically targeted cardiovascular indices (e.g., heart rate variability) and longer interventions may be required to detect physiological change. The study contributes to the psychophysiological gratitude literature by assessing subjective affect and cardiac responding within the same randomized design.

## 1 Introduction

Gratitude has emerged as one of the central constructs in positive psychology because of its consistent associations with psychological well-being, interpersonal functioning, and physical health (Cousin et al., 2021; Wang and Song, 2023). Rather than being understood solely as a transient emotional response to receiving a benefit, gratitude has also been conceptualized as a dispositional tendency, an affective state, and a deliberate psychological practice that facilitates adaptive cognitive appraisal, strengthens social relationships, and promotes resilience in the face of adversity (Cousin et al., 2021; Wood et al., 2010). Since the emergence of positive psychology, gratitude has attracted substantial empirical attention because it represents a relatively simple, inexpensive, and scalable intervention capable of enhancing psychological functioning across both clinical and non-clinical populations. This empirical tradition traces in large part to Emmons and McCullough's (2003) foundational experimental demonstration that instructing participants to count their blessings, relative to counting hassles or neutral daily events, produced heightened well-being across several outcome measures; building on this foundation, numerous subsequent intervention studies have demonstrated that structured gratitude exercises—including gratitude journaling, gratitude letters, and reflective writing—can increase subjective well-being, life satisfaction, optimism, and positive affect while reducing symptoms of depression, stress, and negative affect (Cunha et al., 2019; Datu et al., 2022; Meyer and Stutts, 2024; Predatu et al., 2024; Wang and Song, 2023).

A terminological clarification is warranted at the outset. The present study, consistent with the measurement instrument used, refers throughout to positive and negative *affect* — the broad, valenced feeling states assessed by the Positive and Negative Affect Schedule (PANAS; Watson et al., 1988) — rather than to discrete “emotions” such as appreciation, indebtedness, or guilt. Affect and emotion are related but not interchangeable constructs: consistent with Russell and Barrett's (1999) distinction between core affect and prototypical emotional episodes, affect refers to a general, always-present dimension of subjective feeling (positive or negative in valence), whereas emotion typically denotes a more differentiated, object-directed episode involving a specific eliciting appraisal, has one perceived cause, and is comparatively rare. Because the present design measured only global positive and negative affect via the PANAS, conclusions in this manuscript are restricted to affect; claims about discrete emotional experience are drawn only from the cited literature (Russell and Barrett, 1999) and are explicitly marked as such.

The beneficial effects of gratitude are generally interpreted within the framework of the broaden-and-build theory of positive emotions (Fredrickson, 2001), which proposes that positive emotional experiences expand individuals’ momentary thought–action repertoires and facilitate the accumulation of enduring psychological, social, and physiological resources. From this perspective, gratitude is not merely an emotional outcome but also an adaptive regulatory process capable of promoting flexible cognition, effective coping, and long-term well-being (Fredrickson, 2001). Individuals experiencing gratitude are more likely to reinterpret stressful events constructively, perceive greater social support, and engage in prosocial behaviors, thereby creating upward spirals of psychological functioning (Fredrickson, 2001). Consequently, gratitude interventions have become among the most widely investigated positive psychology interventions because they are relatively easy to implement while demonstrating reliable improvements in emotional well-being across diverse settings (Cunha et al., 2019; Datu et al., 2022; Regan et al., 2023).

Although the psychological benefits of gratitude have been well documented, considerably less is known about its underlying physiological mechanisms. Increasing attention has therefore shifted from asking whether gratitude improves well-being to understanding how these affective changes become biologically embedded (Cousin et al., 2021; Wang and Song, 2023). This transition reflects a broader movement within positive psychology toward integrating subjective emotional experiences with objective physiological indicators. Rather than relying exclusively on self-report measures, researchers have increasingly argued that physiological indices provide complementary evidence regarding the regulatory processes through which gratitude may influence health outcomes (Cousin et al., 2021; Schwerdtfeger et al., 2025). In particular, cardiovascular functioning has become one of the primary biological systems investigated because emotional regulation is closely intertwined with autonomic nervous system activity (Laborde et al., 2017).

The autonomic nervous system continuously regulates cardiovascular activity through dynamic interactions between sympathetic and parasympathetic branches (Laborde et al., 2017; Quintana et al., 2016). Emotional experiences alter this balance, producing measurable changes in heart rate, blood pressure, vascular resistance, cardiac output, and heart rate variability (Laborde et al., 2017). Positive emotions have generally been associated with greater parasympathetic activation, faster physiological recovery from stress, and improved cardiovascular regulation (Blascovich and Tomaka, 1996; Laborde et al., 2017). Nevertheless, evidence suggests that these physiological responses are substantially more complex than initially assumed: rather than producing uniform reductions in cardiovascular activation, positive emotions appear to influence different physiological pathways depending on contextual, interpersonal, and motivational factors (Blascovich and Tomaka, 1996; Gallagher et al., 2021). Accordingly, gratitude should not automatically be expected to produce a straightforward reduction in heart rate but may instead modify broader patterns of cardiovascular adaptation during stressful or emotionally salient situations (Blascovich and Tomaka, 1996).

### 1.1 Why examine heart rate?

Because a null result for any single cardiovascular index is only informative if the index itself is well justified, it is necessary to state explicitly why HR — rather than the broader and more diffuse notion of “cardiovascular function” — was selected as the physiological outcome in this study, and to distinguish HR from that broader construct throughout. Cardiovascular function encompasses multiple, only partially correlated systems (heart rate, heart rate variability, blood pressure, cardiac output, and total peripheral resistance among them; Laborde et al., 2017; Quintana et al., 2016), and a change (or absence of change) in HR cannot be treated as evidence for or against gratitude’s physiological effects in general. HR was chosen here specifically because (a) it is one of the few cardiovascular indices that can be sampled repeatedly, non-invasively, and unobtrusively across a brief laboratory session using low-cost equipment such as a fingertip pulse oximeter (Laborde et al., 2017), making it practical for a five-occasion within-session design; and (b) prior work has linked gratitude — as both a trait and an experimentally elicited state — to cardiovascular reactivity during acute psychological stress, including reactivity indexed partly through HR and related hemodynamic measures (Enko et al., 2021; Gallagher et al., 2021; Gu et al., 2022; Leavy et al., 2023; Schwerdtfeger et al., 2025). This literature does not converge on a directional prediction — some studies report gratitude-related buffering of cardiovascular stress responses (Gu et al., 2022), others report associations with recovery rather than reactivity (Leavy et al., 2023), and others find effects concentrated in indices other than HR itself, such as total peripheral resistance, cardiac output, or heart rate variability (Enko et al., 2021; Gallagher et al., 2021; Jackowska et al., 2016; Newman et al., 2021; Redwine et al., 2016) — which is precisely why HR was treated in the present study as a specific, narrow index worth testing empirically rather than as a construct with an obvious expected direction of effect (see Hypothesis 3 below).

A related question, distinct from the reactivity-based rationale above, is why a session-averaged HR value of this kind should be expected to say anything about mental or physical health more broadly, rather than being an arbitrary convenience measure. The clearest existing basis for treating HR *level* as health-relevant comes from a large epidemiological literature linking resting heart rate to cardiovascular and all-cause mortality risk in the general population: elevated resting HR predicts increased cardiovascular disease mortality independent of traditional risk factors (Cooney et al., 2010), and a meta-analysis of prospective cohort studies totaling over 1 million participants found a graded, dose-dependent association between higher resting HR and both cardiovascular and all-cause mortality (Zhang et al., 2016). This literature is the reason resting HR level, and not only HR reactivity to a stressor, is of interest to health researchers. However, it must be stated plainly that this epidemiological literature concerns resting HR measured under standardized, typically supine or seated rest conditions and often averaged across repeated clinical visits or years of follow-up — a very different measurement context from a single 15-min average taken while participants were actively engaged in psychoeducation, reflection, and writing. The present HR measure is therefore better described as a task-embedded session average than as a clinical resting HR, and the present study does not claim that a change in this specific measure would carry the same health-predictive meaning as the resting HR values used in the mortality literature. This gap between the two measures is acknowledged explicitly as a limitation of drawing any health or well-being inference from the present HR data (see Limitations), and the epidemiological literature is cited here only to establish why average HR level is a construct of health interest in principle, not to claim that the present 15-min average has been validated as a health indicator in its own right.

This rationale is further supported by a broader, non-gratitude literature on structured and expressive writing, which has directly measured cardiovascular activity before, during, and after writing tasks — and which is worth reviewing in more detail because it reveals that writing does not affect HR in a single, uniform direction. In an early and influential demonstration, Pennebaker et al. (1987) found that HR and systolic blood pressure dropped below baseline following the disclosure of traumatic, but not superficial, topics, consistent with a relaxation-like autonomic pattern following deep emotional disclosure. In contrast, Sloan et al. (2007), who experimentally varied the specific writing instructions given to participants, found that emotional disclosure could instead produce an *increase* in HR relative to control writing, indicating that the direction of the HR response depends on precisely how the writing task is instructed and structured rather than following automatically from “writing about something emotional”. Using a longer-term follow-up design, Bourassa et al. (2017) randomly assigned recently separated adults to narrative expressive writing, traditional expressive writing, or a control writing condition and examined average HR, HR variability, and blood pressure several months later, finding effects concentrated in HR variability and blood pressure rather than in average HR itself — a pattern that anticipates the present study’s own null result for HR specifically. Using a within-session, real-time psychophysiological approach closely analogous to the present design, Jacques et al. (2020) recorded electrocardiographic and electrodermal activity continuously *during* an expressive-writing task versus a neutral-writing control task and found writing-related differences in physiological responding while participants were actively writing, underscoring that cardiovascular measurement taken during, rather than only before and after, a writing task can be informative. McGuire et al. (2005) similarly randomly assigned adults with elevated blood pressure to expressive-writing or control conditions and found writing-related change in heart rate variability and blood pressure over a multi-month follow-up, again with effects concentrated outside of HR itself. Most directly relevant to the present active-control design, Seeley et al. (2016) compared an emotional-processing writing task with a structurally matched “fact control” writing task — closely paralleling the present study’s gratitude-versus-ordinary-routine writing comparison — and found no significant main effect of condition on heart rate variability, with effects instead emerging only as a function of individual differences in emotional-approach coping. Taken together, this literature indicates that HR has been reported to decrease, increase, or remain unaffected following writing interventions depending on the specific instructions, content, and timescale involved (Bourassa et al., 2017; McGuire et al., 2005; Pennebaker et al., 1987; Sloan et al., 2007), which is precisely why the present study did not adopt a directional hypothesis for HR and instead treated it as an empirical question. The methodologically closest precedent, Seeley et al. (2016), offers an independent basis for a null or condition-independent main effect on a cardiac index following a brief, content-manipulated writing task, consistent with the present study’s own null result for HR.

Recent gratitude research reflects this conceptual shift by increasingly incorporating physiological outcomes alongside affective measures. Early investigations primarily focused on demonstrating improvements in subjective well-being, whereas more recent studies have attempted to identify cardiovascular correlates of gratitude through laboratory paradigms, ecological momentary assessment, and experimental intervention designs (Cousin et al., 2021; Newman et al., 2021; Schwerdtfeger et al., 2025). This progression illustrates the evolution of the field from descriptive demonstrations of affective benefits toward mechanistic explanations integrating psychological and physiological processes. Across the last decade, gratitude research has gradually expanded from examining self-reported affect alone to investigating autonomic cardiovascular responding during stress, recovery, and interpersonal interaction (Gallagher et al., 2021; Gu et al., 2022).

Despite this progress, empirical findings concerning cardiovascular outcomes remain considerably less consistent than findings concerning affective outcomes. A substantial body of literature demonstrates that gratitude interventions reliably increase positive affect and frequently reduce negative affect (Cunha et al., 2019; Datu et al., 2022; Meyer and Stutts, 2024; Regan et al., 2023). However, studies examining physiological responses have produced heterogeneous findings. While some investigations report cardiovascular patterns consistent with improved stress regulation, others observe negligible or null effects on heart rate itself, instead identifying changes in related indices such as total peripheral resistance, cardiac output, or heart rate variability (Enko et al., 2021; Gallagher et al., 2021; Gu et al., 2022; Jackowska et al., 2016; Leavy et al., 2023; Newman et al., 2021; Redwine et al., 2016).

One explanation for these inconsistencies is methodological heterogeneity. Existing studies differ substantially in intervention duration, intervention format, physiological measurement strategy, laboratory context, and outcome selection. Some studies assess resting heart rate, whereas others focus on cardiovascular reactivity, recovery, heart rate variability, cardiac output, or vascular resistance (Enko et al., 2021; Gallagher et al., 2021; Jackowska et al., 2016; Laborde et al., 2017; Redwine et al., 2016). Consequently, direct comparisons across studies become difficult because apparently inconsistent findings may actually reflect differences in the physiological construct measured rather than contradictory evidence regarding gratitude itself. This methodological diversity has become one of the most frequently acknowledged limitations within the contemporary gratitude literature.

Although gratitude interventions consistently demonstrate beneficial effects on affective well-being, the magnitude and durability of these effects vary across intervention formats and participant characteristics. Meta-analytic and systematic review evidence indicates that gratitude exercises generally produce small-to-moderate improvements in positive affect while simultaneously reducing negative affective states such as stress, sadness, and psychological distress (Cunha et al., 2019; Datu et al., 2022; Kirca et al., 2023; Meyer and Stutts, 2024; Wang and Song, 2023). Nevertheless, these improvements are not uniformly observed across all intervention protocols. Factors including intervention duration, delivery mode, baseline psychological functioning, and the choice of outcome measures appear to moderate intervention effectiveness (Kirca et al., 2023; Regan et al., 2023). For example, brief single-session interventions often yield immediate improvements in affective functioning, whereas multi-session interventions may facilitate more stable affective changes over time (Kirca et al., 2023). Similarly, interventions incorporating interpersonal gratitude expression frequently produce stronger psychosocial outcomes than solitary gratitude listing exercises, suggesting that social context constitutes an important determinant of intervention efficacy (Regan et al., 2023).

Importantly, contemporary gratitude research has moved beyond treating affective experience as a simple bipolar construct. Rather than assuming that gratitude merely increases positive affect while reducing negative affect, recent theoretical developments propose that gratitude frequently elicits a more complex *emotional* profile at the level of discrete states, even where global affect measures show a simpler pattern. Layous et al. (2017), for example, demonstrated that gratitude expression may simultaneously evoke pleasant emotions such as appreciation and elevation together with socially oriented negative emotions including indebtedness, obligation, and guilt. Similarly, Walsh et al. (2022) argued that gratitude is not exclusively a positive emotional state but rather a socially embedded emotion that can activate multiple affective systems simultaneously depending on interpersonal context — consistent with the find-remind-and-bind theory of gratitude (Algoe, 2012), which proposes that gratitude evolved specifically to help individuals notice, and then strengthen ties with, responsive relationship partners, giving it an inherently social and multidimensional character rather than that of a simple pleasant feeling. Consequently, gratitude interventions may generate mixed emotional experiences even when overall self-reported affect improves; the present study, which measured only global positive and negative affect, cannot itself speak to this discrete-emotion complexity and instead relies on the cited literature for this point.

This multidimensional perspective offers one possible explanation for the heterogeneous findings reported across gratitude intervention studies more broadly. Investigations employing broad measures of positive and negative affect frequently report increases in positive affect accompanied by reductions in general negative affect (Cunha et al., 2019; Datu et al., 2022; Meyer and Stutts, 2024; Wang and Song, 2023) — a pattern the present study was designed to test directly. Studies assessing discrete emotional experiences, by contrast, often reveal simultaneous activation of positive emotions and socially functional negative emotions, indicating that gratitude may not simply replace negative affect with positive affect at the level of discrete emotion (Layous et al., 2017; Walsh et al., 2022). Such findings challenge overly simplistic assumptions regarding gratitude’s affective consequences and suggest that intervention effects should be interpreted within broader social and cognitive appraisal frameworks.

The growing recognition of gratitude as a multidimensional affective process has implications for understanding its physiological correlates. Affective complexity is plausibly reflected in autonomic nervous system functioning because different appraisals may activate distinct cardiovascular pathways (Blascovich and Tomaka, 1996). Consequently, inconsistent physiological findings may partly arise because gratitude-related affect does not uniformly produce relaxation. Rather, gratitude may facilitate adaptive cardiovascular responding by altering how individuals evaluate and regulate stressful situations instead of simply suppressing physiological activation. This interpretation is consistent with contemporary challenge–threat models of cardiovascular functioning (Blascovich and Tomaka, 1996), which conceptualize physiological responses as reflecting motivational and cognitive appraisal processes rather than generalized arousal alone.

Within this framework, gratitude has increasingly been investigated as a potential regulator of cardiovascular adaptation. Rather than focusing exclusively on resting physiological activity, several recent studies have examined cardiovascular responses during acute stress, interpersonal interaction, and recovery periods. Gu et al. (2022), for instance, demonstrated that gratitude expression may buffer threat-related cardiovascular responses depending on social context, while Gallagher et al. (2021) found that trait gratitude was associated with cardiovascular reactivity to acute psychological stress, including social-support-related pathways. Furthermore, Enko et al. (2021) suggested that cardiovascular responses to gratitude are moderated by individual differences including depressive symptoms and trait gratitude. Leavy et al. (2023) further found that HR reactivity specifically mediated the association between trait gratitude and the likelihood of acute myocardial infarction. Taken together, these findings indicate that gratitude may influence cardiovascular functioning through appraisal-dependent mechanisms that are not restricted to direct reductions in resting heart rate.

### 1.2 Averaging heart rate across five occasions and the temporal Organization of Cardiac Responding

Despite these theoretical advances, heart rate remains one of the least consistently investigated physiological outcomes within the gratitude literature, and existing studies have operationalized cardiovascular functioning using a wide variety of indicators, including heart rate, heart rate variability, blood pressure, cardiac output, and total peripheral resistance (Enko et al., 2021; Gallagher et al., 2021; Jackowska et al., 2016; Redwine et al., 2016). Consequently, studies often examine different physiological processes despite referring broadly to “cardiovascular functioning,” and null findings for one physiological marker cannot necessarily be generalized to others. Future gratitude research should standardize cardiovascular assessment protocols and explicitly distinguish HR reactivity, recovery, and broader hemodynamic responses as separate constructs (Laborde et al., 2017; Quintana et al., 2016).

This concern is directly relevant to the present design, in which HR was assessed at five occasions across the experimental session — baseline, education, reflection, writing, and immediately after the activity — and the mean of these five values was used as the primary HR indicator in the main analyses (see Method). This averaging approach was adopted for two reasons. First, because the gratitude and control tasks unfolded through several qualitatively different phases (psychoeducation, reflection, writing), a single pre–post HR comparison — as used for positive and negative affect — could not capture responding during the intervention itself, whereas an average across all five occasions summarizes cardiac activity across the entire procedure rather than only its endpoints. Second, averaging provided a single, simply interpretable HR indicator for the primary between-groups test, paralleling the structure of the affect analyses and minimizing the number of statistical comparisons. This approach nonetheless has a clear limitation, acknowledged here directly: an average across five occasions can obscure a genuine reactivity-and-recovery trajectory (e.g., an initial rise followed by a return to baseline) that a repeated-measures or change-score analysis would detect, and a null result for the average does not rule out a null-summing pattern of opposite-signed changes across occasions (see Limitations). The repeated-measures ANOVA reported in the Results, which models all five occasions and their interaction with group, was included specifically to address this concern and to test for differences in the *pattern* of HR responding, not only its average level.

Another important limitation concerns the frequent separation of psychological and physiological outcomes in the broader literature. Although numerous studies report improvements in positive or negative affect, physiological variables are often omitted entirely or examined in separate investigations; conversely, laboratory studies focusing on cardiovascular indices frequently provide limited assessment of discrete affective change. This methodological separation makes it difficult to determine whether affective improvements and physiological regulation occur simultaneously or whether they represent relatively independent consequences of gratitude interventions. Accordingly, several recent reviews have highlighted the need for multimodal experimental designs capable of assessing both subjective affective responses and physiological regulation within the same intervention protocol (Cousin et al., 2021; Wang and Song, 2023).

Finally, intervention characteristics themselves may contribute to inconsistent findings. Gratitude interventions differ considerably with respect to duration, intensity, delivery format, and control conditions. Many studies employ brief interventions lasting only a single session or several days, making it difficult to determine whether physiological adaptation requires repeated practice. Furthermore, active control conditions frequently differ from gratitude interventions in motivational engagement or cognitive demands, thereby complicating causal interpretations regarding gratitude-specific effects (Kirca et al., 2023). Contemporary reviews and meta-analyses of gratitude interventions (Cousin et al., 2021; Kirca et al., 2023; Wang and Song, 2023) therefore recommend longer intervention protocols incorporating repeated physiological assessment and carefully matched control conditions to isolate the unique contribution of gratitude practice.

Despite these methodological recommendations, relatively few experimental studies have simultaneously evaluated changes in positive affect, negative affect, and cardiovascular functioning following a gratitude intervention within a single experimental protocol. Existing studies have generally prioritized either affective outcomes or physiological indicators, thereby limiting a comprehensive understanding of the mechanisms through which gratitude exerts its effects. As a result, it remains unclear whether immediate improvements in affective functioning are accompanied by concurrent physiological changes or whether these responses follow partially independent pathways. This gap is particularly evident in studies employing brief gratitude interventions, where affective outcomes have been reported more consistently than cardiovascular outcomes. Collectively, the current literature suggests that additional randomized experimental studies integrating subjective and physiological indicators are necessary to clarify the immediate psychophysiological effects of gratitude interventions.

Building upon these limitations, the present study adopts a multimodal experimental approach by simultaneously examining changes in positive affect, negative affect, and heart rate following a brief gratitude intervention, using an *active* control condition matched in structure to the gratitude condition (education, reflection, and writing about ordinary daily and yearly routines) rather than a passive or no-treatment control. Unlike previous investigations that have predominantly relied on either self-report measures or isolated physiological indicators, the current study integrates affective and cardiovascular assessments within the same experimental design. This approach allows for a more comprehensive evaluation of whether the affective benefits of gratitude are paralleled by measurable physiological responses, thereby addressing one of the methodological gaps repeatedly identified in recent gratitude research.

In addition, the present study focuses specifically on the immediate effects of a single-session gratitude intervention. Although brief gratitude exercises have repeatedly demonstrated beneficial effects on affective well-being, their capacity to influence cardiovascular functioning remains uncertain because previous findings have been heterogeneous and methodological approaches have varied considerably across studies. By employing pre- and post-intervention assessments within both an experimental and an active control group, the present study provides a rigorous test of whether a brief gratitude exercise produces concurrent psychological and physiological changes.

Accordingly, the primary aim of the present study was to investigate the immediate effects of a brief gratitude intervention on positive affect, negative affect, and heart rate among university students using a randomized controlled experimental design. Based on previous theoretical and empirical findings, it was hypothesized that participants in the gratitude condition would demonstrate (H1) greater increases in positive affect and (H2) greater reductions in negative affect compared with participants in the active control condition, and (H3) a different pattern of heart-rate responding than participants in the active control condition, without a directional prediction as to which condition would show a higher or lower heart rate. Because heart rate relates to gratitude in an index- and context-dependent manner rather than through a uniform reduction (Enko et al., 2021; Gallagher et al., 2021; Gu et al., 2022; Leavy et al., 2023), Hypothesis 3 was deliberately left non-directional rather than specifying a particular direction of change: (H3) the gratitude and control conditions would differ in their *pattern* of heart-rate responding across the five occasions of the experimental session (baseline, education, reflection, writing, post-activity). These hypotheses were developed to examine whether the affective benefits of gratitude are accompanied by concurrent physiological changes, thereby contributing to the growing literature on the psychophysiological mechanisms underlying gratitude interventions.

## 2 Method

### 2.1 Design

The present study employed a randomized controlled pretest–posttest experimental design with one between-subjects factor (group: gratitude intervention vs. active control) and one within-subjects factor (time: pre-intervention vs. post-intervention) for the affective outcomes, and a five-occasion within-subjects factor for heart rate. Randomized controlled experimental designs are considered the gold standard for evaluating the causal effects of psychological interventions because random assignment minimizes systematic differences between groups and reduces selection bias; the inclusion of repeated measurements before and after the intervention further allows researchers to examine both baseline equivalence and intervention-related changes over time (Kazdin, 2021; Shadish et al., 2002). Participants were randomly assigned to either the gratitude intervention group or the active control group following completion of the baseline assessment, ensuring that each participant had an equal probability of assignment to either condition and thereby increasing the internal validity of the study (Kazdin, 2021; Shadish et al., 2002).

### 2.2 Participants

Participants were recruited through voluntary announcements distributed to undergraduate students at a university in Türkiye. Students who expressed interest in participating were invited to the laboratory and enrolled in the study after providing informed consent. Convenience sampling is frequently employed in experimental psychology because it enables standardized administration of laboratory-based interventions under controlled conditions while maintaining adequate internal validity (Kazdin, 2021).

To minimize potential confounding influences on cardiovascular functioning, several exclusion criteria were established prior to data collection. Individuals were excluded if they (a) reported a history of cardiovascular disease or any condition that could directly influence heart rate, (b) had smoked cigarettes or consumed caffeinated beverages immediately before the experimental session, or (c) reported regular use of medications that could affect cardiovascular or psychological functioning. These criteria were established to reduce physiological variability unrelated to the experimental manipulation and to improve the interpretability of heart-rate measurements (Laborde et al., 2017; Quintana et al., 2016).

Forty-one participants met the eligibility criteria, completed all phases of the experiment, and were included in the final analyses (control group *n* = 20; gratitude group *n* = 21). The two groups did not differ significantly in age, year of study, or regular sports participation (see Results, Preliminary Analyses). Gender distribution differed descriptively between groups, *χ*^2^(1) = 3.40, *p* = 0.065, and is reported with exact group-level counts below; the possible confounding role of this descriptive imbalance is discussed explicitly in the Results and Limitations.

### 2.3 Materials

Positive and negative affect were assessed using the Positive and Negative Affect Schedule (PANAS; Watson et al., 1988), a 20-item self-report measure comprising two 10-item subscales — Positive Affect and Negative Affect. Each item is a single adjective (e.g., “interested,” “upset”) rated on a 5-point scale indicating the extent to which the respondent currently feels that way, with subscale scores computed as the sum of the corresponding 10 items (range 10–50 per subscale). The PANAS was administered immediately before and immediately after the intervention/control activity to assess pre–post change in each condition.

A manipulation-check measure was administered immediately after the intervention/control activity to confirm that the gratitude condition successfully elicited a stronger subjective experience of gratitude than the control condition. This measure consisted of five author-constructed items, each rated on a 7-point scale (1 = *strongly disagree* to 7 = *strongly agree*), reflecting the item content and dimensions commonly used in state-gratitude manipulation checks in the gratitude-intervention literature: (1) “I currently feel grateful”; (2) “I am currently more aware of the positive things in my life”; (3) “The reflection/writing task I just completed evoked a feeling of gratitude or thankfulness in me”; (4) “The reflection/writing task I just completed was personally meaningful to me”; and (5) “I currently feel positive and at peace.” Item scores were summed to yield a total manipulation-check score ranging from 5 to 35, consistent with the means reported below (gratitude condition *M* = 27.38; control condition *M* = 19.90).

Heart rate (HR) was assessed using a CONTEC CMS50F fingertip pulse oximeter (CONTEC Medical Systems Co., Ltd.), a non-invasive device equipped with an electronic data-storage function that detects pulse rate optically and reports pulse-rate values with a resolution of 1 bpm (manufacturer-specified measurement range 30–250 bpm; accuracy ±2 bpm or ±2%, whichever is greater). The device was operated in Auto Record mode: once the pulse signal had stabilized after the sensor was placed on the participant’s finger, the device automatically initiated recording, and recording was automatically terminated and stored as a discrete data segment when the finger was removed from the sensor. HR values were therefore captured through the device’s own electronic recording rather than read and manually transcribed by the experimenter, minimizing transcription error. Stored recordings were subsequently transferred from the device to a computer using the manufacturer’s data-transfer software for statistical analysis.

Because the CMS50F reports pulse rate (beats per minute) rather than beat-to-beat RR intervals, the outcome assessed in the present study is pulse rate/heart rate specifically, not heart rate variability (HRV); the two are not used interchangeably anywhere in this manuscript, and the present design cannot speak to HRV-based conclusions (Laborde et al., 2017; Quintana et al., 2016).

### 2.4 Ethical approval

This study was approved by the Ethics Committee of Ibn Haldun University prior to data collection and was conducted in accordance with the ethical principles of the Declaration of Helsinki. All participants provided written informed consent before participation and were informed that participation was voluntary and that they were free to withdraw from the study at any time without any negative consequences.

### 2.5 Procedure

The experiment was conducted individually under standardized laboratory conditions to maximize experimental control and minimize environmental influences on both affective and physiological responses. Participants were seated in a classroom-style arrangement with identical seating conditions for each experimental session. Environmental conditions were maintained as constant as possible throughout data collection, and potential external confounding factors, including unnecessary noise, interruptions, and environmental distractions, were minimized to ensure consistency across participants.

Upon arrival at the laboratory, participants were welcomed by the researcher and provided with information regarding the general procedures of the study. After written informed consent had been obtained, eligibility criteria were reviewed to confirm that participants met the inclusion requirements and none of the predefined exclusion criteria.

Participants first completed the baseline PANAS. Baseline physiological assessment was then initiated. Heart rate was assessed on five separate occasions during the experimental session, coded as T1–T5: T1 (baseline, prior to the intervention/control activity), T2 (immediately following the psychoeducational introduction — gratitude-focused for the gratitude condition, or the parallel neutral introduction for the control condition), T3 (during the reflection period — gratitude-focused reflection or ordinary-routine reflection, respectively), T4 (immediately following the writing task), and T5 (at the close of the session). The mean of these five occasions (“average HR”) was used as the primary heart-rate indicator in the main analyses, and all five occasions were additionally modeled as a repeated-measures factor to test for differences in the pattern of responding (see Introduction and Results). Both the gratitude and the control condition followed this identical five-occasion HR assessment schedule.

Each of the five HR occasions was captured automatically by the pulse oximeter (see Materials) at the point in the protocol described above (T1–T5), and this schedule was identical across participants and across the gratitude and control conditions, addressing the concern that measurement timing might otherwise have varied idiosyncratically across participants.

Following completion of the baseline assessment, participants were randomly assigned to either the gratitude intervention or the active control condition.

Gratitude condition. Participants first received a brief psychoeducational session introducing (a) what positive psychology is and its main subfields, (b) what gratitude specifically is — and is not, (c) the psychological characteristics associated with grateful individuals, and (d) a prompt inviting participants to consider their own daily awareness of gratitude. Following this session, participants spent several minutes reflecting on, and then writing about, people, life events, or personal experiences for which they genuinely felt grateful, in that order — first thinking, then writing — intended to activate gratitude-related cognitions before and during the writing task. The instructions given to participants, translated from the original Turkish, were as follows: “Let’s think about what positive psychology is, its subfields, and what gratitude specifically is and is not. Let’s think about the psychological characteristics of people who experience gratitude. Are we aware, in our daily life, of the things we are grateful for? Let’s think about this, and then write about it”.

Active control condition. Participants assigned to the control condition completed a task matched to the gratitude condition in overall structure and duration but directed toward ordinary, non-gratitude content and explicitly excluding any psychological framing. Participants were instructed to think about, and then write about, their ordinary daily life and yearly routines, described in the same sequential (think-then-write) structure as the gratitude condition, without reference to gratitude, meaning, or any other psychological dimension. The instructions given to participants, translated from the original Turkish, were as follows: “Let’s think about our daily life and our yearly routines — without addressing their psychological dimension at all — just our routines and our experiences. What do we do during the day? What do we do across the year as the seasons change? Let’s think about these in order, and then write about them”.

Writing duration was standardized and identical across both conditions: all participants were given exactly 15 min to write, received verbal time-remaining reminders during the writing period (e.g., at fixed intervals before time was up), and were asked to stop writing simultaneously at the end of the 15-min period, regardless of condition.

Immediately after the writing task, all participants completed the post-intervention PANAS, and the final (fifth) HR measurement was obtained using the same standardized procedure. The manipulation-check measure (see Materials) was administered last, after the post-intervention PANAS, so that participants’ PANAS responses were not immediately preceded by the manipulation-check items. This pretest–posttest protocol enabled simultaneous examination of immediate changes in both affective functioning and cardiovascular activity following the intervention, thereby addressing a methodological limitation frequently identified in previous gratitude research — namely, the tendency to assess psychological and physiological outcomes separately.

The study was not double-blinded: participants were necessarily aware of the content of the activity they completed (gratitude versus ordinary-routine writing), and the experimenter administering the sessions and recording HR was not blind to condition. This is acknowledged explicitly as a limitation below.

## 3 Results

### 3.1 Preliminary analyses

The dataset was first screened for missing values, data-entry inconsistencies, and implausible scores. Complete data were available for all 41 participants across the affect and heart-rate variables (control *n* = 20; gratitude *n* = 21).

During data screening, a previously created positive-affect difference variable was found to contain calculation errors for nine participants; none of the analyses reported below used this pre-existing difference-score column. Positive- and negative-affect change scores were instead recalculated directly from the original pretest and posttest values. The negative-affect and heart-rate difference variables were consistent with their corresponding raw scores.

The experimental groups did not differ significantly in age (control: *M* = 20.60, SD = 1.73; gratitude: *M* = 20.67, SD = 1.32), Welch’s *t* (35.50) = 0.14, *p* = 0.891. No statistically significant differences were observed in year of study, *χ*^2^ (3) = 1.45, *p* = 0.694, or regular participation in sports, *χ*^2^ (1) = 0.60, *p* = 0.440.

Gender distribution differed descriptively between groups: the control group comprised 17 female and 3 male participants, whereas the gratitude group comprised 21 female and 0 male participants, *χ*^2^ (1) = 3.40, *p* = 0.065. This difference did not reach conventional statistical significance, but with a modest sample and unbalanced cell sizes the test has limited power to detect a true gender imbalance, so it should not be read as ruling one out. Because gender has been associated with self-reported affect and gratitude in other samples, this descriptive imbalance is treated as a plausible, uncontrolled confound when interpreting the affect findings below and is discussed further in the Limitations. Given the small expected frequencies in some demographic cells (notably the 0-count male cell in the gratitude group), these categorical comparisons should be interpreted cautiously.

Baseline positive-affect scores did not differ between the control group (*M* = 30.00, SD = 7.60) and the gratitude group (*M* = 30.38, SD = 7.69), Welch’s *t* (38.95) = 0.16, *p* = 0.874. Baseline negative affect was also comparable between the control group (*M* = 22.35, SD = 5.19) and gratitude group (*M* = 23.67, SD = 8.40), Welch’s *t* (33.59) = 0.61, *p* = 0.548. Baseline heart rate was somewhat higher descriptively in the gratitude group (*M* = 86.90, SD = 14.76) than in the control group (*M* = 81.30, SD = 12.78), but this difference was not statistically significant, Welch’s *t* (38.66) = 1.30, *p* = 0.201.

Shapiro–Wilk tests indicated that the positive-affect change scores in the gratitude condition departed from normality, *W* = 0.86, *p* = 0.006, and negative-affect change scores in the control condition also showed some deviation from normality, *W* = 0.89, *p* = 0.022. The remaining primary change-score distributions did not show statistically significant departures from normality. Levene’s tests did not indicate significant heterogeneity of variance for positive-affect, negative-affect, or heart-rate change scores, all *p*s > 0.08. Given the approximately balanced group sizes and the robustness of the mixed ANOVA to moderate non-normality, parametric analyses were retained as the primary analyses; baseline-adjusted ANCOVA models and nonparametric sensitivity analyses were also conducted to assess the robustness of the findings.

Because the target sample size could not be fully reached, a sensitivity power analysis (rather than an *a priori* analysis) is reported here to characterize what the achieved sample could and could not detect, following recommended practice for studies whose recruitment fell short of an original *a priori* target (Faul et al., 2007). For an independent-samples comparison with *n*₁ = 20 and *n*₂ = 21 (*df* = 39) at α = 0.05, two-tailed, the study had 80% power to detect a between-group effect of Cohen’s *d* ≥ 0.90 — a large effect. Applying this benchmark to the observed effects, achieved power was approximately 60% for the positive-affect change-score comparison (Hedges’ *g* = 0.71), approximately 85% for the negative-affect change-score comparison (Hedges’ *g* = 0.96), and only approximately 14% for the heart-rate first-versus-last comparison (Hedges’ *g* = 0.28). This pattern indicates that the study was reasonably well powered to detect the negative-affect effect that was in fact observed, moderately powered for the positive-affect effect, and clearly underpowered to detect an effect of the small magnitude observed for heart rate — meaning the null HR finding should be interpreted as inconclusive with respect to small true effects, rather than as evidence that no true effect exists (see Limitations).

### 3.2 Manipulation check

To confirm that the gratitude intervention successfully elicited a stronger subjective experience of gratitude than the control activity, participants completed the manipulation-check measure described in the Materials section immediately after the intervention/control activity. Participants in the gratitude intervention group reported significantly higher manipulation-check scores (*M* = 27.38, SD = 6.18) than participants in the control condition (*M* = 19.90, SD = 5.38), Welch’s *t* (38.70) = 4.14, *p* < 0.001, Hedges’ *g* = 1.26. The nonparametric Mann–Whitney test yielded the same conclusion, *U* = 350.00, *p* < 0.001. These results indicate that the gratitude manipulation successfully produced a stronger gratitude-related experience in the experimental group than the ordinary-routine control task.

### 3.3 Effects of the gratitude intervention on positive affect

A 2 (Group: gratitude vs. control) × 2 (Time: pretest vs. posttest) mixed ANOVA was conducted to examine changes in positive affect. The main effect of time was statistically significant, *F* (1, 39) = 6.37, *p* = 0.016, partial *η*^2^ = 0.140, and — critically — the Group × Time interaction was also statistically significant, *F* (1, 39) = 5.33, *p* = 0.026, partial *η*^2^ = 0.120, indicating that the increase in positive affect differed reliably between groups.

Descriptively, participants in the control group showed essentially no change in positive affect from pretest (*M* = 30.00, SD = 7.60) to posttest (*M* = 30.10, SD = 7.72; mean change = 0.10, *SD* = 3.19), and the within-group change in the control group was not statistically significant, paired *t* (19) = 0.14, *p* = 0.890. Participants in the gratitude condition, by contrast, showed a clear increase from pretest (*M* = 30.38, SD = 7.69) to posttest (*M* = 33.43, SD = 7.83; mean change = 3.05, SD = 4.78), and this within-group increase was statistically significant, paired *t* (20) = 2.92, *p* = 0.008. To state this plainly, in response to a request for clarity here: the control group showed *no* reliable change in positive affect, whereas the gratitude group showed a reliable increase — the significant interaction reflects this qualitative difference (present vs. absent effect), not merely a difference in the size of two otherwise similar changes (Figure 1).

**Figure 1:**
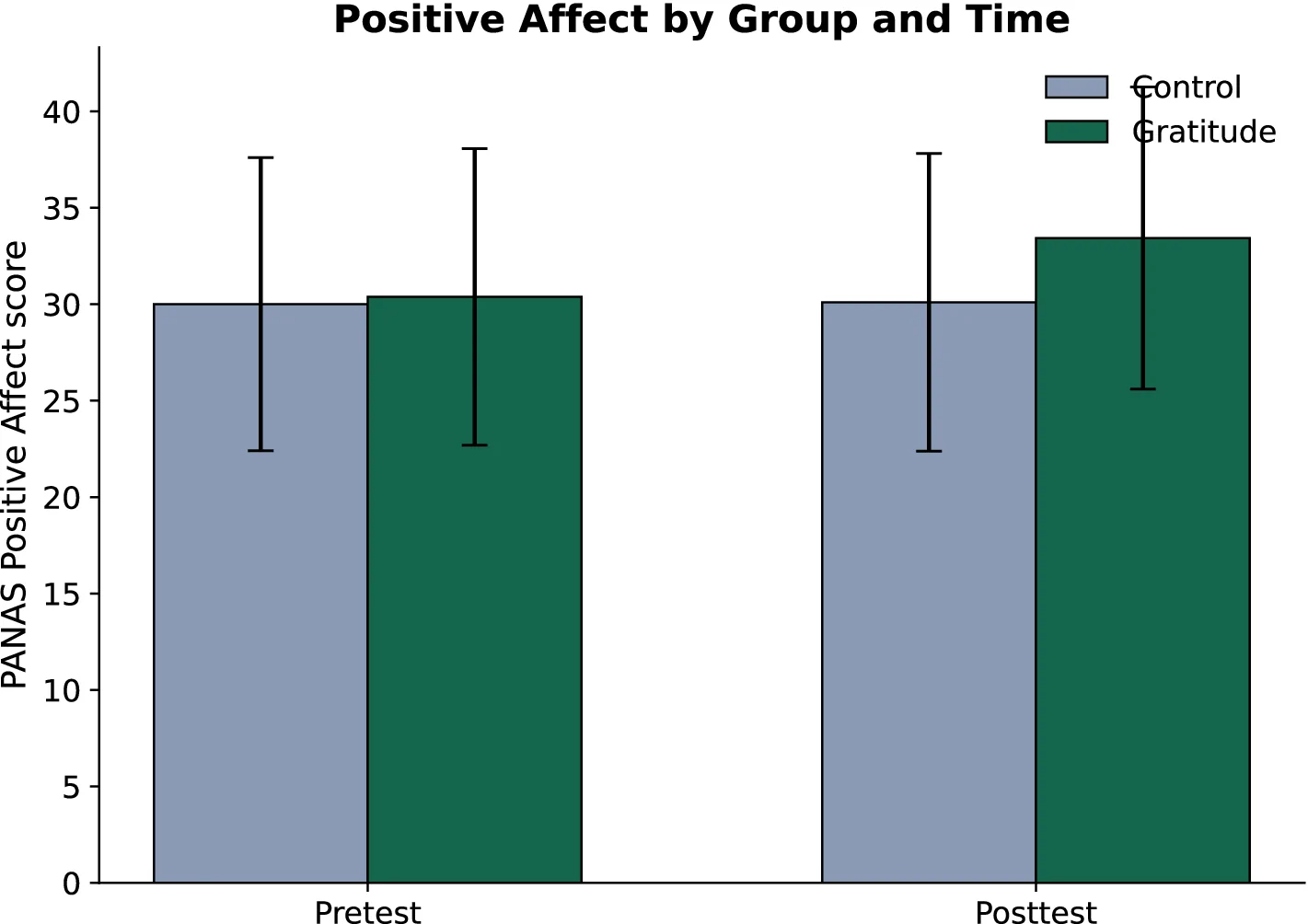
Positive affect (PANAS) at pretest and posttest by condition. Bars show group means; error bars show ±1 SD as reported in the text.

A Welch-adjusted comparison of the raw change scores confirmed a significant group difference, *t* (35.04) = 2.33, *p* = 0.026, Hedges’ *g* = 0.71. A baseline-adjusted ANCOVA, with post-intervention positive affect as the outcome and baseline positive affect as a covariate, confirmed the adjusted effect of experimental condition, *B* = 3.00, 95% CI [0.45, 5.54], *F* (1, 38) = 5.68, *p* = 0.022; the baseline-by-group interaction was not significant, *F* (1, 37) = 0.36, *p* = 0.551, supporting the homogeneity-of-regression-slopes assumption. The Wilcoxon signed-rank test also showed a significant pretest-to-posttest increase within the gratitude group, *p* = 0.007; however, the Mann–Whitney comparison of positive-affect change scores between groups did not reach significance, *U* = 280.50, *p* = 0.066. Thus, although the primary mixed ANOVA, ANCOVA, parametric change-score analysis, and within-group nonparametric test converged in supporting the intervention effect, the between-group nonparametric sensitivity test was less conclusive.

Overall, Hypothesis 1 was supported: the gratitude intervention was associated with a greater increase in positive affect than the active control condition, although this conclusion should be interpreted in light of the modest sample size, the non-normal distribution of change scores in the gratitude condition, and the descriptive gender imbalance between groups noted above.

### 3.4 Effects of the gratitude intervention on negative affect

A second 2 × 2 mixed ANOVA was conducted for negative affect. The main effect of time was significant, *F* (1, 39) = 46.38, *p* < 0.001, partial *η*^2^ = 0.543, and the Group × Time interaction was also statistically significant, *F* (1, 39) = 9.86, *p* = 0.003, partial *η*^2^ = 0.202.

Negative affect declined in both conditions, but to differing degrees. In the control group, scores decreased from *M* = 22.35 (SD = 5.19) at pretest to *M* = 19.30 (SD = 5.97) at posttest (mean reduction = 3.05, *SD* = 4.48), a statistically significant within-group reduction, paired *t* (19) = −3.05, *p* = 0.007. In the gratitude condition, scores decreased from *M* = 23.67 (SD = 8.40) to *M* = 15.29 (SD = 6.93; mean reduction = 8.38, SD = 6.21), a substantially larger and also statistically significant reduction, paired *t* (20) = −6.19, *p* < 0.001. Unlike the positive-affect result, here *both* groups changed reliably, but the gratitude group’s reduction was significantly larger — a difference in magnitude of a shared effect, rather than a present-versus-absent effect as was the case for positive affect (Figure 2).

**Figure 2:**
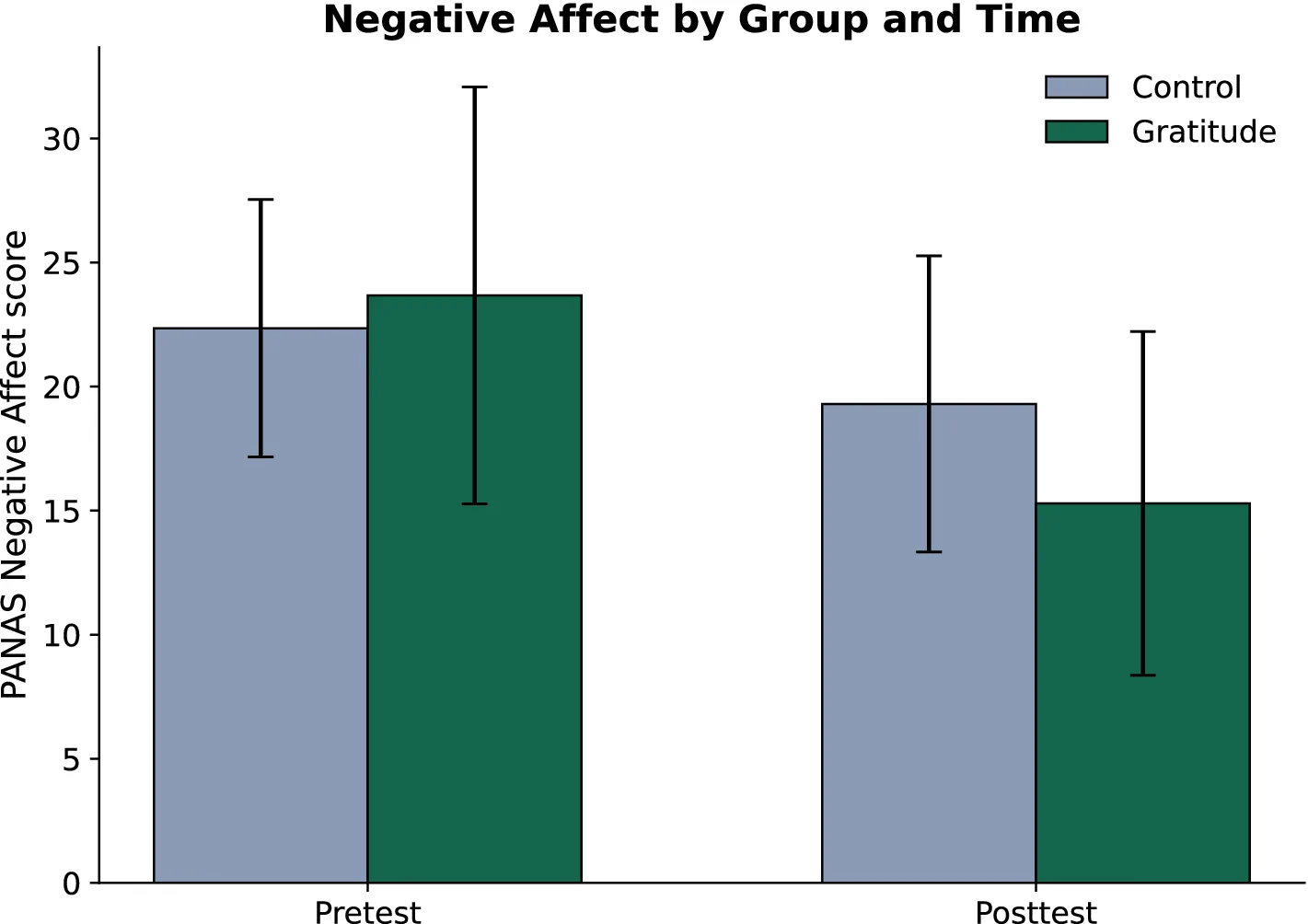
Negative affect (PANAS) at pretest and posttest by condition. Bars show group means; error bars show ±1 SD as reported in the text.

The between-group difference in change scores was statistically significant, Welch’s *t* (36.39) = −3.16, *p* = 0.003, Hedges’ *g* = −0.96. The ANCOVA controlling for baseline negative affect confirmed a significant intervention effect, *B* = −4.84, 95% CI [−7.90, −1.78], *F* (1, 38) = 10.23, *p* = 0.003; the baseline-by-group interaction was not significant, *F* (1, 37) = 0.79, *p* = 0.381. The nonparametric sensitivity analysis also supported the finding, *U* = 96.00, *p* = 0.003. These converging results support Hypothesis 2.

### 3.5 Effects of the gratitude intervention on heart rate

Heart rate was examined across the five successive measurement occasions using a 2 (Group) × 5 (Measurement Occasion) mixed ANOVA. The main effect of measurement occasion was not statistically significant, *F* (4, 156) = 0.28, *p* = 0.892, partial *η*^2^ = 0.007, indicating that heart rate did not show a systematic overall increase or decrease across the experimental procedure in either group. The Group × Measurement Occasion interaction was also nonsignificant, *F* (4, 156) = 0.75, *p* = 0.560, partial *η*^2^ = 0.019, indicating that the *pattern* of heart-rate change across the five occasions did not differ significantly between the gratitude and control conditions — the specific test of Hypothesis 3 as reframed in the Introduction.

The between-subjects group effect approached, but did not attain, conventional statistical significance, *F* (1, 39) = 3.21, *p* = 0.081, partial *η*^2^ = 0.076. This reflects generally higher heart-rate values in the gratitude group across the whole experimental session rather than a differential *response* to the intervention; because the gratitude group also had a numerically (though not significantly) higher baseline heart rate, this between-subjects effect should not be interpreted as evidence that the intervention itself increased heart rate (Figure 3).

**Figure 3:**
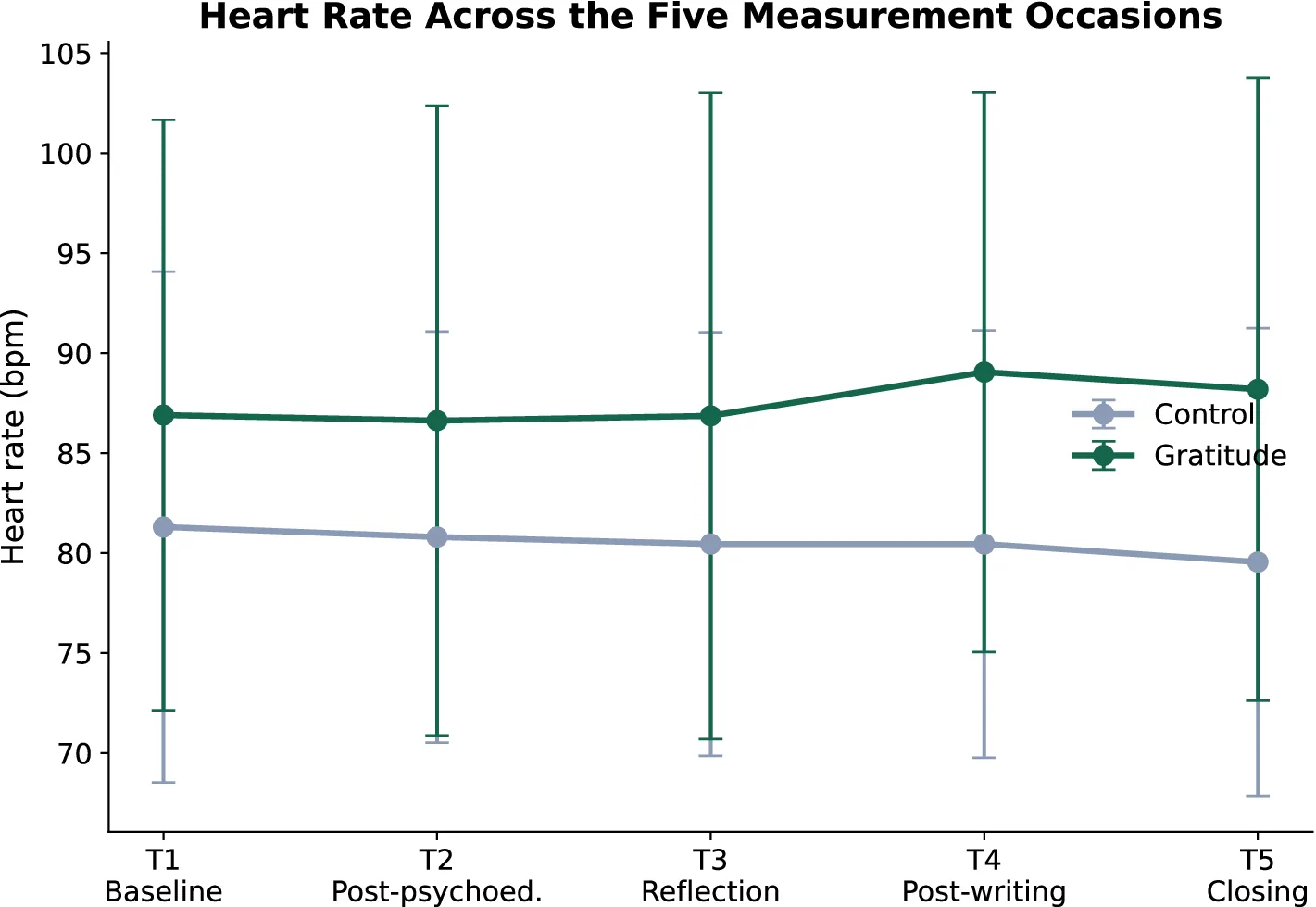
Heart rate (M ± SD) at each of the five measurement occasions (T1 baseline, T2 post-psychoeducation, T3 reflection, T4 post-writing, T5 closing), by condition.

When only the first and final heart-rate measurements were compared, the control group showed a mean reduction of 1.75 beats per minute (SD = 7.82), whereas the gratitude group showed a mean increase of 1.29 beats per minute (SD = 12.65); this difference was not statistically significant, Welch’s *t* (33.58) = 0.93, *p* = 0.359, Hedges’ *g* = 0.28. The Mann–Whitney sensitivity analysis was likewise nonsignificant, *U* = 227.00, *p* = 0.666. Accordingly, Hypothesis 3 — that the gratitude and control conditions would differ in their pattern of HR responding — was not supported by either the repeated-measures interaction or the first-versus-last comparison (Table 1).

**Table 1:** Summary of hypothesis tests

Hypothesis	Key statistic	Conclusion
H1: Gratitude increases positive affect	Group × Time: *F* (1, 39) = 5.33, *p* = 0.026, partial *η*^2^ = 0.120	Supported
H2: Gratitude reduces negative affect	Group × Time: *F* (1, 39) = 9.86, *p* = 0.003, partial *η*^2^ = 0.202	Supported
H3: Groups differ in HR pattern across 5 occasions	Group × Occasion: *F* (4, 156) = 0.75, *p* = 0.560, partial *η*^2^ = 0.019	Not supported

## 4 Discussion

The present study investigated the immediate psychological and physiological effects of a brief gratitude intervention using a randomized controlled pretest–posttest design with an active control condition. Consistent with Hypotheses 1 and 2, participants who completed the gratitude-writing intervention demonstrated significantly greater increases in positive affect and significantly greater reductions in negative affect than participants in the active control condition. In contrast, and consistent with the non-directional framing of Hypothesis 3, the intervention did not produce a significantly different pattern of heart-rate responding across the experimental session. These findings suggest that even a single-session gratitude intervention can produce rapid improvements in self-reported affect relative to a content-matched active control task, whereas heart rate specifically — one narrow index among several cardiovascular measures — did not detectably change over the same brief period.

The observed increase in positive affect is consistent with a substantial body of literature demonstrating that gratitude interventions reliably enhance positive affective functioning across both clinical and non-clinical populations (Cunha et al., 2019; Datu et al., 2022; Meyer and Stutts, 2024; Wang and Song, 2023). Although the present intervention lasted only one session, participants reported significantly higher positive affect immediately following the gratitude-writing exercise relative to the control group, which showed no reliable within-group change, suggesting that gratitude can rapidly shift affective experience without requiring prolonged practice. This is consistent with previous experimental work on brief gratitude activities (Kirca et al., 2023; Regan et al., 2023), which similarly documents that even short, single-session gratitude exercises can produce measurable affective change relative to comparison conditions.

The findings are also consistent with Fredrickson's (2001) broaden-and-build theory of positive emotions, which proposes that positive emotional experiences broaden individuals’ momentary thought–action repertoires and contribute to the accumulation of enduring psychological resources. From this perspective, gratitude represents more than a pleasant affective experience; it functions as a cognitive-affective process that encourages individuals to reinterpret daily experiences through a more adaptive lens (Fredrickson, 2001). During the writing exercise, participants were required to recall and elaborate on meaningful experiences for which they felt grateful; such reflection plausibly redirected attention away from routine stressors and toward positive interpersonal experiences, consistent with the observed increase in positive affect immediately after the intervention. The present study did not assess longer-term psychological resources, so this interpretation should be treated as consistent with, rather than a direct test of, the upward-spiral component of the broaden-and-build framework.

Importantly, the increase in positive affect observed in the present study was accompanied by a simultaneous reduction in negative affect, and this reduction occurred in both conditions but was significantly larger in the gratitude condition. This pattern is consistent with previous findings suggesting that gratitude interventions influence affective functioning through more than one pathway rather than by increasing pleasant affect alone (Datu et al., 2022; Kirca et al., 2023). Participants in both groups showed some reduction in negative affect across the session, which may reflect factors such as repeated measurement, growing familiarity with the laboratory environment, or general engagement in a structured writing task; nonetheless, the decrease was significantly greater among those who completed the gratitude intervention, suggesting that gratitude-specific content produced a substantially larger affective benefit above and beyond these shared, non-specific factors.

The reduction in negative affect may reflect several complementary psychological mechanisms proposed in the broader literature. Gratitude has been proposed to facilitate positive cognitive reappraisal by encouraging individuals to focus on supportive relationships, personal strengths, and meaningful life experiences instead of immediate stressors or daily frustrations (Fredrickson, 2001; Kirca et al., 2023). Such shifts in attentional focus may reduce affective responses associated with worry, frustration, sadness, or psychological distress. Gratitude may also increase perceived social connectedness and interpersonal support, both of which have been associated with lower levels of negative affective experience in the broader well-being literature (Cousin et al., 2021; Wang and Song, 2023). Consequently, the present findings provide additional evidence that a brief gratitude intervention can simultaneously strengthen positive affect while more strongly buffering against negative affect than an active control task.

At the same time, the present affect-level findings should not be interpreted as evidence that gratitude simply replaces negative emotion with positive emotion at the level of discrete, differentiated emotional states — a stronger claim than the present design can support. Contemporary conceptualizations increasingly emphasize that gratitude is a socially embedded and emotionally complex experience: previous research has demonstrated that gratitude may simultaneously evoke appreciation, elevation, indebtedness, obligation, and even guilt depending on interpersonal context (Layous et al., 2017; Walsh et al., 2022). Because the present study measured only global positive and negative *affect* via the PANAS, and not discrete emotions, it cannot speak directly to this multidimensional emotional profile; the present findings are limited to demonstrating a net improvement in global affect, alongside a positive-affect manipulation check confirming that the intervention was subjectively experienced as more gratitude-inducing than the control task. Any claims about discrete-emotion complexity in this discussion are attributable to the cited literature rather than to the present data.

With respect to Hypothesis 3, the gratitude intervention did not produce a significantly different pattern of heart-rate responding relative to the active control condition. Although this null result might initially seem to sit awkwardly next to the clear affective findings, it is compatible with the broader psychophysiological gratitude literature reviewed in the Introduction. Previous studies have reported heterogeneous cardiovascular findings, with some investigations documenting effects concentrated in heart rate variability, cardiac output, vascular resistance, or stress recovery rather than in heart rate itself (Enko et al., 2021; Gallagher et al., 2021; Gu et al., 2022; Jackowska et al., 2016; Leavy et al., 2023; Newman et al., 2021; Redwine et al., 2016; Schwerdtfeger et al., 2025). Consistent with the framing established in the Introduction, the absence of a significant HR effect in the present study should not be interpreted as evidence that gratitude has no physiological correlates in general — a claim the present design was never positioned to test. It indicates, more narrowly, that this specific cardiac index, measured across this specific five-occasion protocol in healthy young adults, did not differ significantly between conditions.

One explanation concerns the complexity of autonomic nervous system regulation. Heart rate represents the combined influence of sympathetic and parasympathetic activity and is affected by numerous physiological and psychological factors, including respiration, posture, anticipation, cognitive effort, and individual differences in cardiovascular functioning (Laborde et al., 2017; Quintana et al., 2016). Consequently, relatively subtle changes in autonomic regulation following a brief gratitude intervention may not necessarily produce a detectable alteration in overall heart rate. More sensitive physiological indices, particularly heart rate variability, are generally considered more informative indicators of parasympathetic regulation and affective flexibility because they capture beat-to-beat variability rather than overall cardiac frequency (Laborde et al., 2017; Quintana et al., 2016). Several contemporary reviews have therefore recommended that future gratitude research prioritize heart rate variability and additional hemodynamic measures alongside, rather than instead of, heart rate when investigating psychophysiological mechanisms.

The characteristics of the present intervention may also help explain the absence of a significant heart-rate effect. Participants completed a single, standardized 15-min gratitude-writing exercise, and physiological responses were assessed across this brief session. Although this design was appropriate for examining immediate affective changes, cardiovascular adaptation may require repeated engagement with gratitude practices over longer periods. Affective experiences can shift rapidly following brief cognitive interventions, whereas physiological adaptation often develops more gradually through repeated activation of regulatory processes (Cousin et al., 2021; Wang and Song, 2023). It is therefore plausible that measurable cardiovascular changes would emerge following multi-session interventions or longitudinal gratitude practice rather than after a single experimental session, and the analytic decision to average HR across five within-session occasions (discussed in the Introduction and Limitations) may itself have limited sensitivity to a brief, session-internal reactivity-and-recovery pattern.

Another possible explanation relates to the characteristics of the sample. Participants consisted of healthy university students who were not selected based on elevated stress levels, affective difficulties, or cardiovascular risk. Healthy young adults typically demonstrate relatively efficient autonomic regulation under resting laboratory conditions, leaving limited opportunity for measurable physiological change within a single brief session. Gratitude interventions may produce stronger physiological effects among individuals experiencing chronic stress, anxiety, depression, or cardiovascular dysregulation, where greater potential for physiological adaptation exists (Redwine et al., 2016). Future studies including more diverse and clinically relevant populations may therefore provide a more comprehensive understanding of the physiological consequences of gratitude interventions.

Methodological considerations should also be acknowledged, and are addressed in full in the Limitations section below rather than repeated here: the fingertip-pulse-oximeter measurement of HR offers considerably less physiological information than electrocardiography-based HRV or continuous cardiovascular monitoring, and the present design captured only immediate, single-session responses rather than delayed recovery or longer-term adaptation. Future research combining electrocardiographic recordings, heart rate variability, blood pressure, and additional biomarkers - including dispositional-gratitude biomarker work outside the acute-intervention literature (Hartanto et al., 2022) - may provide a more detailed understanding of the biological pathways through which gratitude influences health (Cousin et al., 2021; Redwine et al., 2016; Schwerdtfeger et al., 2025).

An important contribution of the present study lies in its integration of subjective affective outcomes and objective physiological assessment within a single randomized experimental design using an active, content-matched control condition. Much of the existing gratitude literature has focused primarily on either psychological outcomes or physiological indicators, making direct comparisons between affective and biological responses difficult. By simultaneously examining positive affect, negative affect, and heart rate, the present study provides a more comprehensive evaluation of the immediate effects of gratitude than would be available from either outcome alone. The findings suggest that affective and cardiovascular responses may follow partially independent trajectories, with psychological improvements emerging more rapidly, or being more detectable with the present sample size and design, than measurable heart-rate change. This distinction contributes to the growing recognition that positive psychological interventions should be evaluated using multidimensional outcome measures rather than relying exclusively on either subjective or physiological indicators.

From an applied perspective, the present findings support the use of brief gratitude interventions within educational, organizational, and mental health settings for their affective benefits specifically. Because the intervention required minimal resources and produced immediate improvements in self-reported affect relative to a matched active control task, gratitude-writing exercises may represent an accessible component of well-being promotion programs, psychoeducational workshops, university counseling services, and preventive mental health initiatives. Claims about physiological or cardiovascular health benefits should be made more cautiously, since the present study’s only cardiovascular index did not show a significant effect; additional research is required before recommending brief gratitude writing as a cardiovascular intervention specifically.

Overall, the present findings indicate that a brief gratitude intervention produced clear, immediate benefits for self-reported affect relative to a matched active control condition, while heart rate — one specific and narrowly defined cardiovascular index — did not show a corresponding group difference within the same brief session. Rather than contradicting previous research, this pattern is consistent with a psychophysiological gratitude literature in which cardiovascular effects, when present, are often concentrated in indices other than resting or session-averaged heart rate, or require longer or more intensive interventions to emerge (Enko et al., 2021; Gallagher et al., 2021; Jackowska et al., 2016; Leavy et al., 2023; Redwine et al., 2016; Schwerdtfeger et al., 2025). Continued investigation using longer intervention protocols, more sensitive cardiovascular measures such as heart rate variability, and multimodal assessment strategies will further clarify how gratitude contributes to both psychological well-being and physical health (Cousin et al., 2021; Wang and Song, 2023).

## 5 Limitations

Several limitations should be considered when interpreting the findings of the present study.

First, the sample size was relatively small (*N* = 41) relative to the study’s original recruitment target, which reduced statistical power to detect subtle physiological changes in particular. As reported in the Results (sensitivity power analysis), the achieved sample provided approximately 80% power only for effects of Cohen’s *d* ≥ 0.90, meaning the study was well powered to detect the negative-affect effect that was observed but clearly underpowered (~14% power) to detect an effect of the magnitude observed for heart rate; the null heart-rate finding should therefore be read as inconclusive for small true effects rather than as positive evidence of no effect. Replication with larger samples would provide more precise estimates of the magnitude of both affective and physiological intervention effects.

Second, participants consisted exclusively of healthy university students recruited through convenience sampling from a single university in Türkiye. Consequently, the findings cannot be readily generalized to broader populations, including older adults, individuals with chronic health conditions, or clinical populations experiencing elevated psychological distress. Gratitude interventions may produce different physiological responses among individuals with higher baseline stress levels or impaired autonomic regulation. Future studies should therefore recruit more diverse samples across different age groups, cultural contexts, and clinical settings.

Third, the present study examined only the immediate effects of a single-session gratitude intervention. Although brief interventions provide valuable information regarding short-term affective changes, physiological adaptations may require repeated practice over extended periods. Longitudinal studies incorporating multiple intervention sessions and follow-up assessments would provide a more comprehensive understanding of the durability of both affective and physiological effects.

Fourth, although writing duration was standardized at 15 min for both conditions, with time-remaining reminders and a simultaneous stop for all participants, the reflection and psychoeducation phases preceding writing were not governed by an identical fixed-duration protocol; some naturalistic variability in the length of these earlier phases across participants cannot be ruled out and should be reported precisely (e.g., minimum, maximum, and mean duration for the psychoeducation and reflection phases) in future work using this protocol.

Fifth, regarding the heart-rate assessment specifically, the primary analysis relied on the mean of five within-session HR occasions. As discussed in the Introduction, this averaging approach can obscure a genuine reactivity-and-recovery trajectory across the session; although the repeated-measures ANOVA reported in the Results directly tested for such a pattern and likewise found no significant Group × Occasion interaction, a design using continuous or more finely time-locked HR recording, together with *a priori* segmentation into reactivity and recovery phases (rather than five discrete occasions), would provide a more sensitive test of gratitude-related cardiac responding. It also remains unclear from the original protocol whether each of the five HR occasions was captured at an identical, fixed time point within its corresponding phase for every participant and every condition; this should be reported precisely in any future version of this manuscript or in subsequent studies using this design.

Sixth, heart rate was measured using a fingertip pulse oximeter because of its practicality and ease of administration under laboratory conditions. Although this method provides a reasonable estimate of momentary heart rate, it does not capture more subtle indices of autonomic nervous system regulation. Heart rate variability, electrocardiographic recordings, blood pressure, cardiac output, and additional physiological biomarkers may offer more sensitive indicators of gratitude-related physiological processes. Future research should therefore adopt multimodal psychophysiological assessment strategies to better understand the biological mechanisms underlying gratitude interventions.

Seventh, affective outcomes were assessed exclusively through the self-report PANAS measure. Although self-report measures remain the standard approach for assessing subjective affective experience, they are susceptible to response biases, including social desirability and demand characteristics; because the study was not double-blinded, participants’ awareness of their assigned condition may have contributed to demand-characteristic effects on self-reported affect specifically. Combining self-report instruments with behavioral, observational, or physiological measures of affective regulation may provide a more comprehensive evaluation of intervention effectiveness in future work. Relatedly, one item of the manipulation-check measure — “I currently feel positive and at peace” — overlaps conceptually with the constructs assessed by the PANAS. The manipulation check was administered last, after the post-intervention PANAS (see Procedure), which means the post-intervention PANAS scores used to test Hypotheses 1 and 2 could not themselves have been contaminated by exposure to this item. The reverse possibility remains open, however: because participants are known to respond in ways that are consistent across closely spaced self-report measures, their manipulation-check ratings — particularly on this overlapping item — may have been influenced by the affect they had just reported on the PANAS, rather than purely reflecting the gratitude manipulation itself. This would not bias the primary affect findings but could inflate the apparent size of the group difference on the manipulation check reported in the Results. Future work should use manipulation-check items with less semantic overlap with the PANAS to rule out this possibility.

Eighth, although participants were randomly assigned to experimental conditions, gender distribution was not balanced across groups (control: 17 female, 3 male; gratitude: 21 female, 0 male), *χ*^2^ (1) = 3.40, *p* = 0.065. This descriptive imbalance did not reach conventional statistical significance, but given the small sample, it cannot be ruled out as a contributing factor to the observed group differences in self-reported affect: because the control group contained all 3 of the study’s male participants and the gratitude group was entirely female, and gender has in other work been associated with self-reported affect or gratitude responsiveness, some portion of the affect findings could in principle reflect this compositional difference rather than the gratitude manipulation alone. Because the present sample size does not permit a well-powered test of gender as a moderator, this possibility can be described but not resolved with the current data; future randomized controlled trials with larger and more demographically balanced samples, and with gender entered as a covariate or moderator, would strengthen the generalizability and interpretability of the findings.

Finally, terminology should be used precisely in interpreting these results: the present study assessed global self-reported *affect* (via the PANAS) and a single cardiovascular index (HR), not discrete “emotion” or “cardiovascular function” as broader constructs; conclusions have been restricted accordingly throughout this revision, and readers should likewise avoid generalizing beyond affect and HR specifically when citing these results.

## 6 Conclusion

The present study examined the immediate psychological and physiological effects of a brief gratitude intervention using a randomized controlled experimental design with an active control condition. Consistent with previous positive psychology research, participants who completed the gratitude-writing exercise demonstrated significantly greater increases in positive affect and greater reductions in negative affect than participants assigned to the active control condition. In contrast, no significant group difference was observed in the pattern of heart-rate responding across the experimental session. This indicates that, within the present single-session design, healthy young-adult sample, and averaged five-occasion measurement of heart rate specifically, the immediate effect of gratitude on this cardiovascular index was not detected — a narrower and more precise conclusion than a general claim that gratitude’s “physiological effects” are less pronounced than its emotional benefits, which the present single-index design cannot support.

These findings contribute to the growing psychophysiological literature by demonstrating that affective and cardiovascular responses to gratitude may not emerge on the same timescale, or may not both be detectable within a brief single-session protocol of this kind. Whereas subjective affective improvements were evident immediately following the intervention relative to an active control task, a corresponding change in heart rate was not observed within the same experimental session. This distinction highlights the importance of evaluating gratitude interventions using both subjective and objective outcome measures, of selecting and reporting physiological indices with an explicit rationale, and of resisting the temptation to generalize a null finding on one narrow index to physiological effects in general.

From a practical perspective, the findings support the use of brief gratitude exercises as accessible interventions for promoting self-reported emotional well-being among university students. Because the intervention required minimal time and resources while producing significant improvements in affective functioning relative to a matched active control condition, gratitude-based activities may be incorporated into university counseling services, psychoeducational programs, and other preventive mental health initiatives — with claims limited to affective, rather than cardiovascular, benefits pending further research.

Future research should investigate whether longer intervention periods, repeated gratitude practice, and more sensitive or better-justified cardiovascular measures — particularly heart rate variability, alongside HR — yield different physiological results, and should standardize task duration and the timing of repeated physiological measurements across participants and conditions. Studies involving larger and more demographically balanced samples, formally reported statistical power, longitudinal follow-up assessments, and multimodal physiological measurement will further clarify the mechanisms through which gratitude influences both psychological well-being and physical health (Cousin et al., 2021; Wang and Song, 2023).

## Data Availability

The raw data supporting the conclusions of this article will be made available by the authors, without undue reservation.
